# Sudden Sensorineural Hearing Loss: A Review

**DOI:** 10.7759/cureus.29458

**Published:** 2022-09-22

**Authors:** Priyanshi Tripathi, Prasad Deshmukh

**Affiliations:** 1 Medicine, Jawaharlal Nehru Medical College, Datta Meghe Institute of Medical Sciences, Wardha, IND; 2 Otolaryngology - Head and Neck Surgery, Jawaharlal Nehru Medical College, Datta Meghe Institute of Medical Sciences, Wardha, IND

**Keywords:** sudden hearing impairment, hearing loss, emergency in e.n.t, covid 19 and hearing loss, sudden sensorineural hearing loss

## Abstract

The objective of this review is to present and evaluate the current views on sudden sensorineural hearing loss. Sudden sensorineural hearing loss is an otolaryngology emergency requiring immediate diagnosis and treatment. The etiology of the majority of cases is unknown. The etiology can be classified into various categories such as autoimmune diseases, infections, functional, metabolic disorders, vascular disorders, traumatic causes, due to toxins, and neurological disorders. We searched the U.S. National Library of Medicine's PubMed database using the terms "sudden S.N.H.L.," "virus and ssnhl," and "sudden deafness," as well as the keywords such as "etiology," "COVID 19, and ssnhl," “epidemiology,” “clinical characteristics,” “management,” “therapy,” and “prognosis."

## Introduction and background

Sudden sensorineural hearing loss (SSNHL) requires immediate diagnosis and treatment. There may be complete or partial loss of hearing; it is defined as an abrupt start of loss in hearing that is more than 30db or more over at least three contiguous audiometric frequencies occurring within 72 hours [1].

SSNHL can present as unilateral or bilateral; the bilateral one in high frequencies is usually symmetrical. The prevalence of SSNHL is estimated to be 5-20 cases per 100,000 individuals annually. Despite extensive study, questions about the origin, proper care, and treatment of individuals with idiopathic SSNHL remain unanswered. Recovery in this SSNHL is not guaranteed as it depends on the cause. However, partial recovery and rarely total recovery have also been documented. Age is one factor that influences hearing recovery following SSNHL. The units of loss in hearing, the frequencies affected, the association with vertigo, and the period between the start and seeking therapy are all factors influencing hearing recovery following SSNHL [2].

Although the etiology behind the majority of SSNHL cases is idiopathic, vascular compromise is the most widely accepted theory among them. The cochlea is supplied by two tiny arteries that are terminal. The cochlea is prone to damage by significant factors such as the small diameter and absence of collateral. Unilateral SSNHL has a clinical appearance comparable to ischemic vascular disorders such as amaurosis fugax and transient ischemic episodes. There are several ideas concerning the pathogenesis of idiopathic SSNHL that include some of the autoimmune causes such as systemic lupus erythematous (SLE), Behçet’s disease, and Cogan’s syndrome. Infections that are related to SSNHL are meningitis, fungal meningitis, AIDS, mumps, syphilis, Lassa fever, mycoplasma, Lyme disease, *Toxoplasma gondii *infection, and certain endocrinologist disorders including diabetes mellitus and hypothyroidism, as well as cancers such as schwannoma vestibule, petrous meningeomas, and myeloma C.P.A [3-7].

There are three possible pathways for how viral infection causes SSNHL. Virus affects the cochlear nerve, soft tissue, or fluid spaces in the cochlea. Under specific conditions, the other process is when the latent virus gets reactivated inside inner ear tissues. The last mechanism includes the immune-mediated hypothesis, which has triggered antibody response due to prior infections that cross-react with the internal ear antigens [3].

Lyme disease and syphilis are the two most prevalent bacterial diseases that cause SSNHL in the United States of America. he infecting agent is spirochete *Borrelia burgdorferi,* which is spread by bite of deer tick. To transmit the bacterium, the tick (infected) needs to be connected to a human host for around two to three days. An increasing erythematous rash (erythema migrans) that lasts approximately two to three weeks without treatment is a common early symptom of this illness. Within the first year of infection, chronic Lyme disease symptoms such as systemic neurologic involvement leading to facial paralysis and asymmetric sensorineural hearing loss (SNHL) might emerge. Rheumatologic diseases such as arthritis, cardiac problems such as atrioventricular block, and central nervous system (CNS) disorders such as chronic meningoencephalitis and fibromyalgia are some other manifestations [4].

Syphilis is caused by *Treponema palladium*, which is a sexually transmitted disease. Syphilis is also called the great copier as it has a broad spectrum of symptoms. Following syphilis infection, the patient presents in the OPD with painless skin lesions on the genitalia called a chance (primary disease). The patient may later show CNS manifestations, which is the stage known as neurosyphilis, which can deliver as otosyphilis, even at this early stage [8].

**Figure 1:**
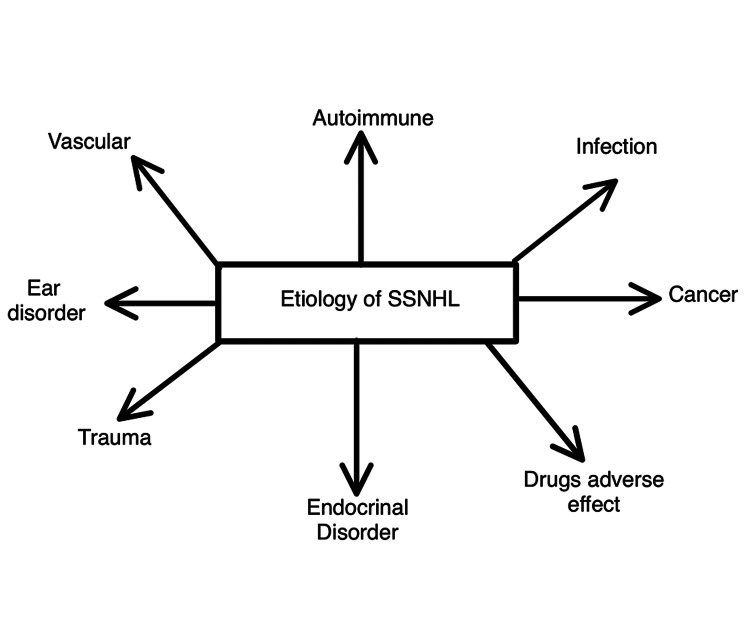
Etiology of SSNHL SSNHL, sudden sensorineural hearing loss

SSNHL is a gene that can be found in autoimmune and thyroid diseases. A sudden hearing loss is linked to autoimmune illnesses, for example, Cogan's syndrome, SLE, temporal arteritis, and Wegener's granulomatosis. SSNHL can be caused by several neoplasms, notably vestibular schwannomas (acoustic neuromas). In individuals with SSNHL, the prevalence of vestibular schwannoma varies from 0% to 48%. SSNHL is linked to many vascular and hematologic diseases [9-12]. Figure 1 represents the etiological summary of SSNHL.

## Review

Diagnosis

SSNHL is characterized by unilateral brief hearing loss or hearing loss that occurs immediately after waking up. A typical ear examination reveals stuffy or full ears, tinnitus, and vertigo as related clinical complaints. A patient's evaluation includes obtaining a history of triggering events, such as a URI (upper respiratory infections) or trauma, the severity of hearing loss, laterality, onset or duration, and accompanying symptoms. The feeling of a complete ear, also known as the stuffy sensation of the ear, should not exclude the doctor from detecting SSNHL and must be distinguished from conductive hearing loss (CHL) to be diagnosed. Tuning fork examinations are a valuable tool for determining the severity and type of hearing loss. The Weber test includes placement of a tuning fork in the bony prominences such as over the glabella, nose bridge, head top, or upon the central upper incisors to determine the kind of loss (with a rubber glove over the handle). The sound will be perceived in the sick ear in CHL, and the sound will be perceived in the standard functional ear in SSNHL. If a tuning fork is not accessible, the Rauch test can be used instead. Solicit a low-pitched hum from the sufferer. The buzz will be heard in the sick ear in CHL and the proper functional ear in SNHL. The Rinne test is used to determine the extent of conductive loss; however, it is ineffective in SSNHL. An audiogram should be obtained as soon as feasible once SNHL has been established by tuning fork testing. Treatment with steroids should begin immediately rather than waiting for an audiogram or a referral [13].

Management

With the help of good history, physical examination, and laboratory investigations such as vestibular tests, audiometry, temporal bone imaging studies, VDRL test for syphilis, diabetes, a blood disorder, thyroid levels, triglycerides, cholesterol levels, and sedimentation rates, one can find out the etiology and treat accordingly. In certain cases, exploratory tympanometry is indicative of ruling out perilymph fistula. As most of the cases are idiopathic, management is empirical and follows the below points [14-18]:

· Bed rest.

· Steroid therapy: Once a morning, dose for about a week of prednisolone 40-60 mg and then lower the amount for about three weeks. The steroid works as an anti-inflammatory that relieves edema.

· Carbogen inhalation: Carbogen consists of 5% carbon dioxide and 95% of oxygen. It effectively improves oxygenation and cochlear blood flow to the inner ear.

· Drugs causing vasodilation (low molecular weight dextran): Its action decreases the viscosity of the blood. It is contraindicated in bleeding disorders and cardiac failure.

· Hyperbaric oxygen therapy: One of the actions of carbogen is increasing the oxygen level in the inner ear; hyperbaric oxygen therapy also works on the exact mechanism of action. It increases the oxygenation level in labyrinthine fluid and improves the cochlea's function.

· Low salt diet and a diuretic: it does not have enough evidence.

Intratympanic (IT) steroid therapy: As the steroid, when given systematically, causes more side effects, IT steroid therapy is uncomfortable to patients, but it increases steroids locally in cochlear fluids.

Treatment

If the etiology of SSNHL is recognized, therapy for the disease should begin immediately. A few studies have found that hearing can improve after treatment for specific diseases such as vestibular schwannoma, mumps, and secondary syphilis [19-21]. Unluckily, therapy given as per the etiologic disease does not restore the hearing to the pre-onset level in severe SSNHL [22]. When asked, 98% of otolaryngologists in the United States of America reported that they used oral steroids to treat idiopathic SSNHL, whereas 8% reported that they utilized IT steroids. Idiopathic SSNHL is improved by corticosteroids, which reduce inflammation and edema in the inner ear. Several therapies are advised for the treatment of idiopathic SSNHL. One of the foundations of this continuing dispute is that idiopathic SSNHL resolves on its own in 45-65% of individuals. Nonrandomized and retrospective studies are often conducted, but many do not specify clinical objectives correctly [23].

Hearing recovery in individuals taking steroids was 78% compared to 38% in placebo patients in a study of 67 participants (double-blind randomized controlled trials) utilizing various corticosteroid regimens. Following attempts to duplicate this trial, conflicting data on the beneficial use of corticosteroids in idiopathic SSNHL were discovered. Many of these experiments have been shown to have methodological flaws [16-17]. Because IT-steroid application yields a more significant amount of steroid level than a systemic injection, at least as seen in guinea pigs and the systemic circulation, IT steroid does not get absorbed; IT corticosteroids are becoming increasingly employed in the treatment of idiopathic SSNHL [14]. According to ongoing research, when hyperbaric oxygen is combined with steroids, that too in high doses, around 59.7% to 86.67% shows positive results, with a significant increase in improvement (seen when there is at least 10 dB gain on average pure tone) in patients’ hearing than with the use of only corticosteroids [24-26].

Prognosis

The illness process, its length, the changes in the cochlear structures, and treatment choices all have a crucial impact on the prognosis of SSNHL owing to a discernible cause (Table 1). In the idiopathic SSNHL, 45%-65% of total patients will restore their pre-loss hearing thresholds independently, with mean improvements of 35 db. Old age (most of the studies took >60 years as old age) has been consistently linked to poorer hearing recovery rates, and if recovery occurs the threshold with which the hearing improves is low among all demographic variables studied.. Presentation to a physician as early as possible, usually less than a week after the onset of SSNHL, also correlates with enhancement in hearing recovery. In contrast, the late presentation has an adverse prognosis. Following audiogram, rates of recovery in hearing in the initial week of onset are 87%, 52% at two weeks, and 10% or less after three months. It has also been observed that SSNHL of smaller duration is likely to recover more irrespective of disease or when the treatment was started. Several studies attempted to build algorithms that integrate numerous prognostic variables to produce a percentage chance of hearing recovery or an odds ratio for patients with various risk factors. The frequency of bilateral SSNHL has been reported to be 2% in numerous studies, and what is more noteworthy is that it occurs synchronously [7,22].

**Table 1:** Prognostic factors in sudden sensorineural hearing loss

Criteria	Better prognosis	Adverse prognosis
Hearing loss	Mild	Severe
Frequency loss	Low and medium	High
Recovery starting duration	Two weeks	Later or never
Age	Young	Old
Vertigo history	Absent	Present
Treatment started	Early	Later

Sudden sensorineural hearing loss and COVID-19

Few case reports were considered when searching the MeSH term for COVID-19 concerning SSNHL [25-29]. All of them have shown a positive relation to COVID-19, either presenting with SSNHL or post-COVID-19 complications. However, still, no apparent cause of it has been found. Although numerous studies are being conducted for other symptoms of COVID-19, this particular symptom of SSNHL and its link to COVID- 19 is not been extensively studied yet. Only about six papers have been published since April 2020 discussing the topic. In the most recent paper, a COVID-19 positive patient with no other etiological factor that can cause SSNHL presented with the complaint of hearing loss, which, on further investigation, was known to be SSNHL. Only oral steroids were prescribed for the treatment, and the hearing was reversible. It is important to note that the patient did not complain of hearing loss before the first visit due to the overwhelming environment and later came up with the complaint. The last case which states SNHL of a patient with SARS-CoV-2 positive was in April 2020 by Sriwijitalai and Wiwanitkit [29]. The following two cases were of COVID-19 positive patients with no prior history of any ear problems developing SSNHL, which was reversible on rapid treatment by IT steroid therapy [27]. In one case report, a 70-year-old man suffering from severe COVID-19 developed right ear deafness and left ear SNHL and was given an IT steroid for the left ear and a cochlear implant for the right-sided hearing impairment. Even after the vaccination with the second dose of the Oxford-AstraZeneca COVID-19 vaccine, a 61-year-old female presented with a history of right-sided ear fullness since four days and complete hearing loss in the same ear since two days. Further investigation with pure tone audiometry depicted SNHL and her treatment was started by giving a combination of glucocorticoids (dexamethasone 8 mg three times per day for three days with tapering over a week) and acetylsalicylic acid. The rationale to use anticoagulant acetylsalicylic acid 100 mg once daily was that as AstraZeneca COVID-19 vaccine (ChAdOx1 nCoV-19, AZD1222, Vaxzevria; Oxford/AstraZeneca) increases the chances of thrombotic event, early usage of anticoagulant agent leads to full recovery after 15 days in this case report [30-32].

## Conclusions

SSNHL is an otolaryngology emergency. The characteristic of SSNHL is rapid onset that is progressive loss over half a day. The male-to-female incidence ratio is equal in SSNHL; the risk factors for SSNHL are noisy environment, pollution, and workers having occupation in which there is constant change of atmospheric pressure such as scuba drivers. Hearing loss is sudden or develops over hours or several days. There are certain predisposing factors for SSNHL, such as the use of ethanol, patient emotional state, fatigue, diabetes mellitus, atherosclerosis, age of the patient, and pregnancy. There are various etiologies that can be subgrouped. Most SSNHL cases are idiopathic, and treatment depends on the cause. If it is idiopathic, around half of patients recover spontaneously without any treatment within two weeks of the onset of hearing loss, although corticosteroids with hyperbaric oxygen are found effective when given within the initial treatment. Although many protocols for treating SSNHL are put forward, they miss adequate evidence. Treatments such as carbogen inhalation, vasodilator drugs, low molecular weight dextran, low salt diet, and diuretics have not proven to be effective. The recovery percentage after one month is inferior; thus, the prognosis is poor. The prognosis is bad if the patient is old, the treatment isw started is late, there is high frequency loss of hearing, and hearing loss associated with vertigo. The ongoing COVID-19 pandemic and its relation with SSNHL has not been researched in depth.
